# Genetic characteristics of pathogenic *Leptospira* in wild small animals and livestock in Jiangxi Province, China, 2002–2015

**DOI:** 10.1371/journal.pntd.0007513

**Published:** 2019-06-24

**Authors:** Cuicai Zhang, Jianmin Xu, Tinglan Zhang, Haiyan Qiu, Zhenpeng Li, Enmin Zhang, Shijun Li, Yung-Fu Chang, Xiaokui Guo, Xiugao Jiang, Yongzhang Zhu

**Affiliations:** 1 State Key Laboratory for Infectious Disease Prevention and Control, National Institute for Communicable Disease Control and Prevention, Chinese Center for Disease Control and Prevention, Beijing, People’ Republic of China; 2 Collaborative Innovation Center for Diagnosis and Treatment of Infectious Diseases, Hangzhou, People’s Republic of China; 3 JiangXi Province Center for Disease Control and Prevention, Nanchang, People’s Republic of China; 4 Southwest university, Chongqing, People’s Republic of China; 5 GuiZhou Province Center for Disease Control and Prevention, Guiyang, People’s Republic of China; 6 Department of Population Medicine and Diagnostic Sciences, College of Veterinary Medicine, Cornell University, Ithaca, New York, United States of America; 7 Department of Microbiology and Immunology, Institute of Medical Science, Shanghai Jiao Tong University School of Medicine, Shanghai, People’s Republic of China; Baylor College of Medicine, UNITED STATES

## Abstract

**Background:**

Leptospirosis is one of the most important neglected tropical bacterial diseases worldwide. However, there is limited information on the genetic diversity and host selectivity of pathogenic *Leptospira* in wild small mammal populations.

**Methodology/Principal findings:**

Jiangxi Province, located in southern China, is a region highly endemic for leptospirosis. In this study, among a total of 3,531 trapped rodents dominated by *Apodemus agrarius* (59.7%), 330 *Leptospira* strains were successfully isolated from six different sites in Jiangxi between 2002 and 2015. Adding 71 local strains from humans, various kinds of livestock and wild animals in Jiangxi, a total of 401 epidemic strains were characterized using 16S rRNA gene senquencing, multilocus sequence typing (MLST) and the microscopic agglutination test (MAT). Among them, the most prevalent serogroup was Icterohaemorrhagiae (61.10%), followed by Javanica (19.20%) and Australis (9.73%); the remaining five serogroups, Canicola, Autumnalis, Grippotyphosa, Hebdomadis and Pomona, accounted for 9.97%. Species identification revealed that 325 were *L*. *interrogans* and 76 were *L*. *borgpetersenii*. Moreover, *L*. *interrogans* was the only pathogenic species in Fuliang and Shanggao and was predominant in Shangrao (95.0%); *L*. *borgpetersenii* was the most common in the remaining three sites. Twenty-one sequence types (STs) were identified. Similarly, ST1 and serogroup Icterohaemorrhagiae were most prevalent in Shangrao (86.0% and 86.4%) and Fuliang (90.4% and 90.4%), ST143 and serogroup Javanica in Shangyou (88.5% and 90.4%) and Longnan (73.1% and 73.1%), and ST105 and serogroup Australis in Shanggao (46.3% and 56.1%). Serogroup Icterohaemorhagiae primarily linked to *A*. *agrarius* (86.9%), serogroup Canicola to dogs (83.3%). There were significant differences in the distribution of leptospiral species/serogroups/STs prevalence across host species/collected locations among the 394 animal-associated strains (Fisher’s exact test, p<0.001).

**Conclusions/Significance:**

Our study demonstrated high genetic diversity of pathogenic *Leptospira* strains from wild small animals in Jiangxi from 2002 to 2015. *A*. *agrarius* was the most abundantly trapped animal reservoir, and serogroup Icterohaemorrhagiae and ST1 were the most dominant in Jiangxi. Significant geographic variation and host diversity in the distribution of dominant species, STs and serogroups were observed. Moreover, rat-to-human transmission might play a crucial role in the circulation of *Leptospirosis* in Jiangxi. Details of the serological and molecular characteristics circulating in this region will be essential in implementing prevention and intervention measures to reduce the risk of disease transmission in China. However, phylogenetic analysis of more *Leptospira* isolates should explore the impact of ecological change on leptospirosis transmission dynamics and investigate how such new knowledge might better impact environmental monitoring for disease control and prevention at a public health level.

## Author summary

Leptospirosis, caused by pathogenic *Leptospira spp*, is one of the most widespread zoonoses. In recent years, human leptospirosis with occasionally fatal infections has been frequently reported in Jiangxi Province, a highly endemic region located in the south of China. However, there is a lack of information on circulating *Leptospira* strains in this province. To identify the etiological characteristics, 401 *Leptospira* from Jiangxi were characterized using serological and molecular typing methods. Serological typing revealed that 61.10% of the isolates belonged to serogroup Icterohaemorrhagiae. Two species, *L*. *interrogans* and *L*. *borgpetersenii*, were identified using 16S rRNA gene sequencing. *A*. *agrarius* may be the main carrier of leptospirosis in this endemic region. Furthermore, the diversity of leptospiral isolates was demonstrated using MLST analysis. ST1, as the most prevalent ST of pathogenic leptospires, was widely dispersed in China. Significant geographic variation and host diversity in the distribution of dominant species, serogroups and STs were found in Jiangxi. This study is the first to demonstrate the distribution of *Leptospira* in domestic and wildlife animals in Jiangxi. This retrospective study represents the longest and largest field epidemiological investigation on the etiological characteristics and genetic diversity of pathogenic *Leptospira* among large wild animal reservoirs and human populations in Jiangxi. A better understanding of the circulating etiological agents and epidemiology of leptospirosis will provide a good starting point for efforts to control and prevent this disease.

## Introduction

Leptospirosis, primarily caused by pathogenic spirochaetes of the genus *Leptospira*, is one of the most widespread and significant zoonotic diseases; it annually causes 1.0 million estimated cases of severe human leptospirosis and 58,900 estimated deaths, as well as great veterinary economic losses worldwide [1, 2]. The genus *Leptospira* contains at least 22 species and more than 300 serovars based on agglutinating lipopolysaccharide (LPS) antigens [3], with 76 serovars and 18 serogroups being reported in China [4]. *L*. *interrogans*, *L*. *borgpetersenii* and *L*. *kirschneri* are the main pathogenic species of leptospirosis in humans and animals worldwide [5, 6]. The clinical symptoms of human leptospirosis range from asymptomatic or mild infection to severe manifestations causing multi-organ dysfunction and even death. In addition to human hosts, pathogenic *Leptospira* also infect a wide range of animals, including domestic mammals (livestock) and wild animals, especially rodents, which are considered the main reservoir for *Leptospira* infections in humans [7]. Humans can be infected through direct contact with infected animals, or indirect contact with water and soil contaminated by the urine of infected animals [8]. Therefore, long-term active surveillance and investigations into the carriage status of animal reservoirs and epidemiological characteristics of animal-associated causative agents and infected individuals will contribute to understanding animal-to-human transmission, field epidemiology, outbreak investigation and source tracking for leptospirosis. Since 1955, *Leptospirosis* has been classified as a nationally notifiable disease in China. During 1955–2010, ten large outbreaks of leptospirosis with incidences rates of more than 10 cases per 100,000 have been previously reported [9]. The Chinese National Notifiable Infectious Disease Surveillance System was established in 2005, in which 25 monitoring sites throughout the whole country were selected to continually survey human cases or animal reservoirs of leptospirosis. The incidence of leptospirosis in the recent decade decreased to 0.1 cases per 100,000 compared to 1.4 cases per 100,000 in the 1990s [9]. Although the incidence of leptospirosis has significantly decreased, small-scale local outbreaks and high prevalence rates were still reported recently in some epidemic regions of China [10].

Jiangxi Province is located in southern China. Historically, this province has been a significant endemic region for leptospirosis. Here, a large number of rivers and lakes, a moist subtropical monsoon climate, abundant rainfall and forest coverage, a wide variety of wild animals, and rice cultivation provide a favorable environment for the broad spread and prevalence of *Leptospira*, as well as a large population of reservoir mammals. Compared to the annual average incidence in China (average 0.0692 cases per 100,000 inhabitants between 2002 and 2015), the annual average human incidence of leptospirosis in Jiangxi was as high as 0.1764 cases per 100,000 inhabitants during the period of 2002–2015, showing that Jiangxi Province was a significant epidemic area of leptospirosis (data available from the annual infectious disease reports of the National Notifiable Infectious Disease Surveillance System in China). Furthermore, the annual average incidence rates in some regions, such as Ganzhou, Yichun, Shangrao and Yingtan city, are markedly higher than those of other regions in Jiangxi Province, while Nanchang and Jingdezhen have relatively lower incidences rates. To date, no detailed long-term studies have focused on the epidemiological characteristics and genetic diversity, including the predominant serogroups or genotypes in wild animals of Jiangxi Province. To investigate potential reservoir populations and the genetic diversity of the strains, a large-scale dataset composed of 401 epidemiological strains from multiple sources in Jiangxi Province was characterized using 16S rRNA gene sequencing and MLST typing. These isolates were primarily obtained from a wide range of wild small animal reservoirs and leptospirosis patients in Jiangxi Province through the National Notifiable Infection Diseases Surveillance System of Leptospirosis over a period of 14 years. To the best of our knowledge, this retrospective study represents the longest and largest field epidemiological investigation on the etiological characteristics and genetic diversity of pathogenic *Leptospira* among dogs, livestock (pigs and cattle) and other small wild animals (*A*. *agrarius*, *R*. *rattoides*, *R*. *norvegicus*, *Rattus flavipectus*, common skunks, *Rana nigromaculata* and so on) and human populations in Jiangxi Province. The detailed serological and molecular characteristics circulating in this region may provide new insights into the epidemiology and guidelines for the control of leptospirosis in China.

## Materials and methods

### Ethics statement

This study and the research protocol were reviewed and approved by the Ethical Committee (Institutional Review Board, IRB) of National Institute for Communicable Disease Control and Prevention, Chinese Centre for Disease control and Prevention (License number: ICDC-2015361). All patients gave written informed consent for participation in this study with their identifiable information, and the legal guardians of young children (less than 12 years of age) provided informed consent on their behalf; in accordance with the Declaration of Helsinki and IRB approval. No livestock were euthanized. Moreover, permission to sample dead livestock of suspected leptospirosis was provided by the owners of these animals. The trapping, handling and euthanasia of wild rodents, *R*. *nigromaculata* and skunks in this study were carried out following the procedures and protocols approved by the Ethical Committee of the National Institute for Communicable Disease Control and Prevention, Chinese Centre for Disease Control and Prevention (License number: ICDC-2015361).

### Monitoring sites in Jiangxi Province

**Fig 1:**
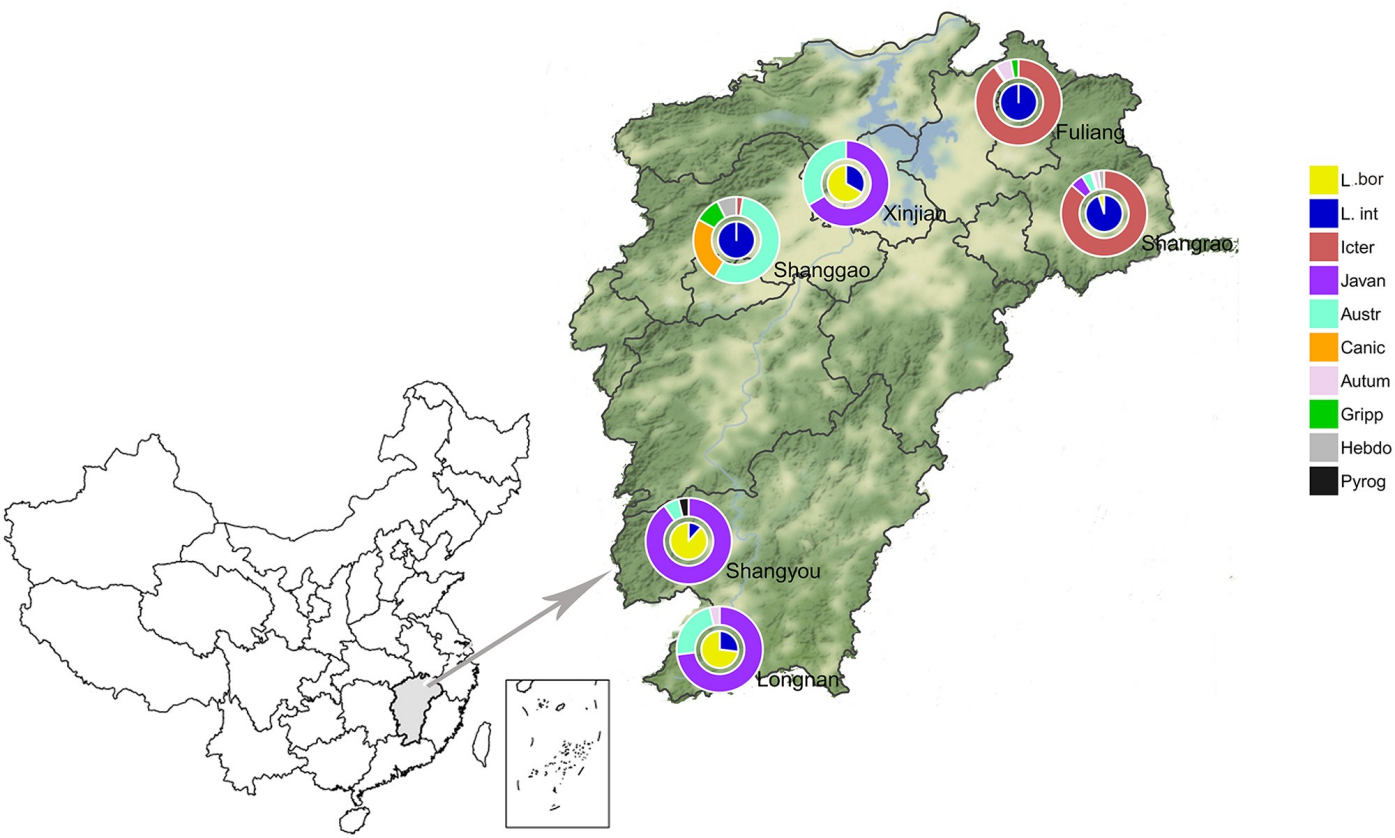
Diversity and geographic distribution of pathogenic *Leptospira* circulating strains in six monitoring sites of Jiangxi Province where animals were trapped. This map was plotted by combination of four different R packages as well as ArcGIS software with version 10.2 (ESRI, USA) and Photoshop CS 8.0.1 software (Adobe Systems, USA), the satellite image was plotted by using ggmap (https://cran.r-project.org/web/packages/ggmap/) and maptools (https://cran.r-project.org/web/packages/maptools/), the pie charts were drawn by using ggplot2 (https://cran.r-project.org/web/packages/ggplot2/) and ggforce (https://cran.r-project.org/web/packages/ggforce/).

In our study, six monitoring sites located in three (designated as Habitat A, B, and C) of five epidemic habitats previously reported in Jiangxi Province based on the incidence of leptospirosis, as well as geographical latitude, longitude, altitude and geomorphic conditions involved in the National Notifiable Infectious Disease Surveillance System [11] (Fig 1). These monitoring sites in Jiangxi represent different ecosystems containing large wild animal populations as well as a biodiversity index. Habitat A, with the lowest incidence rate, includes Xinjian city (average 0.0015 cases per 100,000 between 2002 and 2014) and lies in the northern lower Poyang Lake area of Jiangxi Province. The topography is generally 15–26 meters above sea level. Habitat B has a higher incidence rate; this area includes Fuliang (average 0.0329 cases per 100,000 during 2002–2014), and Shangrao (average 0.2781 cases per 100,000 during 2002–2014), Shanggao (average 0.3162 cases per 100,000 between 2002 and 2014) and lies in the northeastern hilly plain area (100–300 meters above sea level). Habitat C has the highest incidence rate and encompasses Shangyou and Longnan (average 0.2994 cases per 100,000 between 2002 and 2014) with 80.6% of the land covered by forests containing abundant wild animal resources. Habitat C is located in the southwestern South Jiangxi mountain region (1000–1600 meters above sea level). Hence, the diverse topography and ecological conditions of Jiangxi Province made it suitable for investigating the genetic diversity of *Leptospira* from wild small animals in these different ecosystems.

### Rodent trapping and isolation of *Leptospira* in Jiangxi Province

The six trapping sites in Jiangxi were located as follows: Xinjian (28.69 N; 115.82 E), Fuliang (29.4 N; 117.2 E), Shangrao (28.5 N; 117.9 E), Shanggao (28.2 N; 114.9 E), Longnan (24.9 N; 114.8 E) and Shangyou (25.8 N; 114.5 E). Within each locality, rodent trapping was conducted over an area of approximately 10 kilometers squared.

Field rodents were trapped using the Trap-night method from 2007 to 2015 in humid rice field environments known to contain large rodent populations within the framework of the CERoPath project (www.ceropath.org) [12]. The traps were loaded with peanut butter bait in the evening and collected early morning. For each site, 10 trapping lines, consisting of 10 locally hand-made wire traps (approximately 40×12×12 cm) every five meters, were placed during a period of five days and four nights [12]. Field rodents were trapped twice in the wet season among April to June and August to October every year at the same place (using a Global Positioning System receiver). The trapped field rodents and small mammals were identified by genus, species, and gender based on phenotypic characteristics (ears, body, tail, fur color, sex) [13]. The rodent density was calculated using the formula: (Number of rodents trapped each year / Number of total traps successfully placed for each year * 100).

In addition, another 64 strains isolated from dogs, livestock (pigs and cattle) and other wild small animals (*A*. *agrarius*, *R*. *rattoides*, *R*. *norvegicus*, *R*. *flavipectus*, skunk and *R*. *nigromaculata*), serving as potential reservoir animals of leptospirosis in Jiangxi Province; these samples were collected from the same six regions through the same surveillance system between 2002 and 2015 (S1 Table). A total of 7 strains isolated from clinical cases of leptospirosis were collected from urine samples in the local hospitals in Shanggao (S1 Table). Kidney samples from cattle, pigs and dogs were directly collected from the owners of these dead animals in these monitoring sites. Approximately 1 g of fresh kidney samples from the animals or 100–200 μl of whole blood from animals suspected of leptospirosis were cultured in 10 ml of liquid Ellinghausen-McCullough-Johnson-Harris (EMJH) medium (Difco Laboratories, USA) with 5-fluorouracil (Merck, Germany) at 28°C and observed weekly by dark-field microscopy for the presence of *Leptospira* for up to 3 months. Samples with no growth of *Leptospira* after 3 months were considered negative [14].

### Species identification of leptospiral strains isolated from Jiangxi Province

Species identification was performed using 16S rRNA gene sequencing as previously described [15]. A total of 20 accessible *Leptospira* species reference sequences representing pathogenic, intermediate and non-pathogenic *Leptospira* species were obtained from the GenBank database. *Leptonema illini* NCTC 11301T and *Turneriella parva* NCTC 11395T were set as the outgroup (S2 Table) [15, 16]. The sequences of all the 401 *Leptospira* strains isolates from Jiangxi and the 20 representative sequences were compared using Clustal W. A neighbor-joining tree was constructed using Mega software version 5.10 with a bootstrap value of 1,000.

### Serogroup identification of leptospiral strains isolated from Jiangxi Province

Serogroup identification of these leptospiral strains was conducted by MAT against 15 Chinese standard serogroup-specific rabbit antisera from the National Institutes of Food and Drug Control, China, representing the most prevalent pathogenic *Leptospira* serogroup in China. The serogroup scoring the highest MAT titer of the test strain agglutinating 50% of live leptospiral against a given serogroup-specific rabbit antisera was defined as the presumptive corresponding serogroup.

### MLST analysis of isolated strains

MLST was performed using seven housekeeping genes (*glmU*, *pntA*, *sucA*, *tpiA*, *pfkB*, *mreA* and *caiB*) as previously described [17]. The PubMLST *Leptospira* database (http://pubmlst.org/leptospira/) was used for nucleotide analysis. Minimum spanning trees (MST) were applied to determine the relationships among STs through BioNumerics software version 5.10 (Applied Maths, Kortrijk, Belgium). Clonal complexes (CCs) were defined with clustered STs differing by one or two loci and named on the basis of the putative founder ST or the ST associated with the largest number of single-locus variants. Singletons are defined as the STs differing by at least three alleles from other STs. Phylogenetic analysis was performed using the unweighted pair group method with average linkages provided in BioNumerics software version 5.10.

### Statistical analysis

The effects of the prevalent serogroups, STs and species on host-species, collected years and locations were investigated. Fisher’s exact test was used to compare the differences in the distribution of leptospiral prevalence across species, serogroups and STs between small animal species and collected locations among the animal-associated strains. The p-value was computed by Monte Carlo simulation. The statistical Kruskal-Wallis chi-squared test was used to investigate whether there were significant differences in the distributions of leptospiral prevalence across species, serogroups and STs among collected years among the animal-associated strains. All statistical analyses were performed using R software (R version 3.5.1, https://www.r-project.org/) [18], considering a significance level of 0.05.

## Results

### Species of trapped rodents and isolation of leptospiral strains from Jiangxi

A total of 45,144 traps were placed and 3531 field rodents belonging to 9 different species were successfully captured between 2007 and 2015. Species identification of trapped rodents and the number of rodents with positive renal cultures is presented in Table 1. Those species represented most of the small mammal diversity in Jiangxi Province. The density of field rodents was between 4.76–12.56% in Jiangxi Province. The most abundantly trapped species was *A*. *agrarius* (59.7%, 2107/3531), followed by *R*. *rattoides* (23.0%). A total of 330 strains from 5 different species of field rodent were isolated (Table 1). In addition, 34 strains isolated from small wild animals including 10 *A*. *agrarius*, 10 *R*. *rattoides*, 5 *R*. *norvegicus*, 5 *R*. *flavipectus*, 2 *skunks* and 2 *R*. *nigromaculatas*; 30 isolated from livestock including 5 cattle, 1 pig and 24 dogs; and 7 isolated from humans were also collected in the same six sites between 2002–2015. In this study, a total of 401 non-epidemiologically related leptospiral strains collected between 2002 and 2015 in Jiangxi were used (S1 Table). Among these 394 animal-associated strains, *A*. *agrarius* was the main abundantly trapped animal reservoir, accounting for 59.4% (234/394) of carriers identified, followed by *R*. *rattoides* (17.3%).

**Table 1:** Rodent distribution and strains isolated from rodent during 2007–2015 in Jiangxi.

Year	A	B	Rodent density	*Apodemus agrarius*	*Rattus rattoides*	*Rattus flavipectus*	*Rattus norvegicus*	*Mus musculus*	*Rattus confucianus*	*Niviventer fulvescens*	*Cricetulus migratorius*	*Rattus nitidus*	Strains
**2007**	**7834**	**373**	**4.8**	**31/196**	**7/113**	**0/4**	**5/28**	**0/10**	**0/15**	**0/1**	**0/6**	**0/0**	**43**
**2008**	**6512**	**343**	**5.3**	**10/116**	**17/133**	**2/34**	**7/57**	**1/1**	**0/2**	**0/0**	**0/0**	**0/0**	**37**
**2009**	**6091**	**434**	**7.1**	**16/206**	**4/146**	**4/65**	**0/17**	**0/0**	**0/0**	**0/0**	**0/0**	**0/0**	**24**
**2011**	**5341**	**435**	**8.1**	**35/275**	**7/9**	**7/113**	**2/28**	**0/10**	**0/0**	**0/0**	**0/0**	**0/0**	**51**
**2012**	**5470**	**554**	**10.1**	**47/342**	**5/121**	**0/12**	**2/51**	**0/11**	**0/2**	**0/0**	**0/14**	**0/1**	**54**
**2013**	**5205**	**486**	**9.3**	**37/316**	**6/111**	**0/27**	**0/27**	**0/0**	**0/5**	**0/0**	**0/0**	**0/0**	**43**
**2014**	**4956**	**437**	**8.8**	**20/292**	**2/96**	**0/8**	**18/40**	**0/0**	**0/1**	**0/0**	**0/0**	**0/0**	**40**
**2015**	**3735**	**469**	**12.6**	**28/364**	**10/83**	**0/0**	**0/12**	**0/0**	**0/10**	**0/0**	**0/0**	**0/0**	**38**
**Total**	**45144**	**3531**	**7.8**	**224/2107**	**58/812**	**13/263**	**34/260**	**1/32**	**0/35**	**0/1**	**0/20**	**0/1**	**330**

A-Number of total traps placed for each year.

B-Number of rodents trapped for each year.

Rodent density calculated as (B/A * 100).

### Species identification of Jiangxi isolates using 16S rRNA gene sequencing

Using 16S rRNA gene sequencing, two pathogenic species: *L*. *interrogans* and *L*. *borgpetersenii*, were identified among the 401 isolates (S1 Fig). Fisher’s exact test revealed highly significant differences in the distribution of leptospiral species prevalence across host species/collected locations among the 394 animal-associated strains (p<0.001 for all comparisons) (S3 and S4 Tables). *L*. *interrogans* was the predominant species (81.1%), widely represented in all six regions (Fig 1) and identified from humans and a wide range of animals (i.e., 10 host species, S3 Table), while *L*. *borgpetersenii* was only identified from 4 host species (S3 Table). Furthermore, there were some special geographic differences between the circulating pathogenic *Leptospira* species. *L*. *interrogans* was the only species in the two cities of Fuliang and Shanggao and was the predominant species in Shangrao (133/140), while *L*. *borgpetersenii* was the dominant species in the remaining three cities of Shangyou (46/52), Longnan (19/26) and Xinjian (4/6) (S4 Table). The Kruskal-Wallis chi-squared test showed a significant difference in the distribution of leptospiral species prevalence across collected years among the 394 animal-associated strains (χ^2^ = 60.9, df = 9, P < 0.001) (S5 Table). *L*. *interrogans* was present in every year from 2005 to 2015, but *L*. *borgpetersenii* was not present in 2012.

### Serogroup identification of 401 isolated strains obtained from Jiangxi Province from 2002 to 2015

A total of eight serogroups were identified among 401 isolates. Serogroup Icterohaemorrhagiae, as the most frequent serogroup, accounted for 61.1%, followed by Javanica (19.20%) and Australis (9.73%). The remaining five serogroups, Canicola, Autumnalis, Grippotyphosa, Hebdomadis and Pomona, accounted for only 10.0% (S1 Table).

Fisher’s exact test revealed highly significant differences in the distribution of leptospiral prevalence across collected locations among the 394 animal-associated strains (P < 0.001) (S6 Table). Leptospiral serogroup diversity was higher in Shangrao, Shanggao and Fuliang than in Shangyou, Longnan and Xinjian (Fig 1). In addition, there were some significant regional variations in the distribution of the dominant serogroups. Icterohaemorrhagiae was the most common serogroup in Shangrao (121/140) and Fuliang (123/136) and Australis in Shanggao (23/41), while serogroup Javanica was dominant in the remaining three cities of Shangyou (47/52), Longnan (19/26) and Xinjian (4/6) (Fig 1).

Fisher’s exact test revealed highly significant differences in the distribution of leptospiral prevalence across hosts species among these 394 animal-associated strains (P < 0.001) (S7 Table). A couple of serogroups were primarily associated with one or multiple host species. For example, Icterohaemorhagiae was preferentially restricted in *A*. *agrarius* (86.9%), Canicola in dogs (83.3%), and Javanica in *R*. *rattoides* (40.3%) and *R*. *norvegicus* (33.8%).

With the exception of 3 isolates in 2002 and 2 isolates in 2005, there were 31–54 isolates per year between 2006 and 2015 in the 401 isolates. The diversity of serogroups was relatively lower in recent years. An average of 5–7 different serogroups were found per year before 2011. After 2011, only 2 to 4 serogroups were isolated annually. The Kruskal-Wallis chi-squared test showed a significant difference in the proportional prevalence of leptospiral serogroups in different years among the 394 animal-associated strains (χ^2^ = 66.1, df = 9, P < 0.001) (S8 Table). The most prevalent serogroups, Icterohaemorhagiae, Javanica and Australis, were present every year from 2006 to 2015 except 2005, 2012 and 2014. Serogroup Canicola was present in 2006–2007, 2009 and 2011.

### Genetic diversity and population structure analysis of 401 Jiangxi isolates using MLST analysis

In this study, a total of 21 different STs were obtained from 401 pathogenic *Leptospira* isolates in Jiangxi Province (S1 Table). The most prevalent ST was ST1 (235/401), followed by ST143 (72/401), ST105 (24/401), ST37 (15/401) and ST17 (10/401). The remaining 45 isolates belonged to 16 different STs (S1 Table). Additionally, among the 21 STs, only ST209 and ST143 were identified as *L*. *borgpetersenii*; the other STs were identified as *L*. *interrogans*.

**Fig 2:**
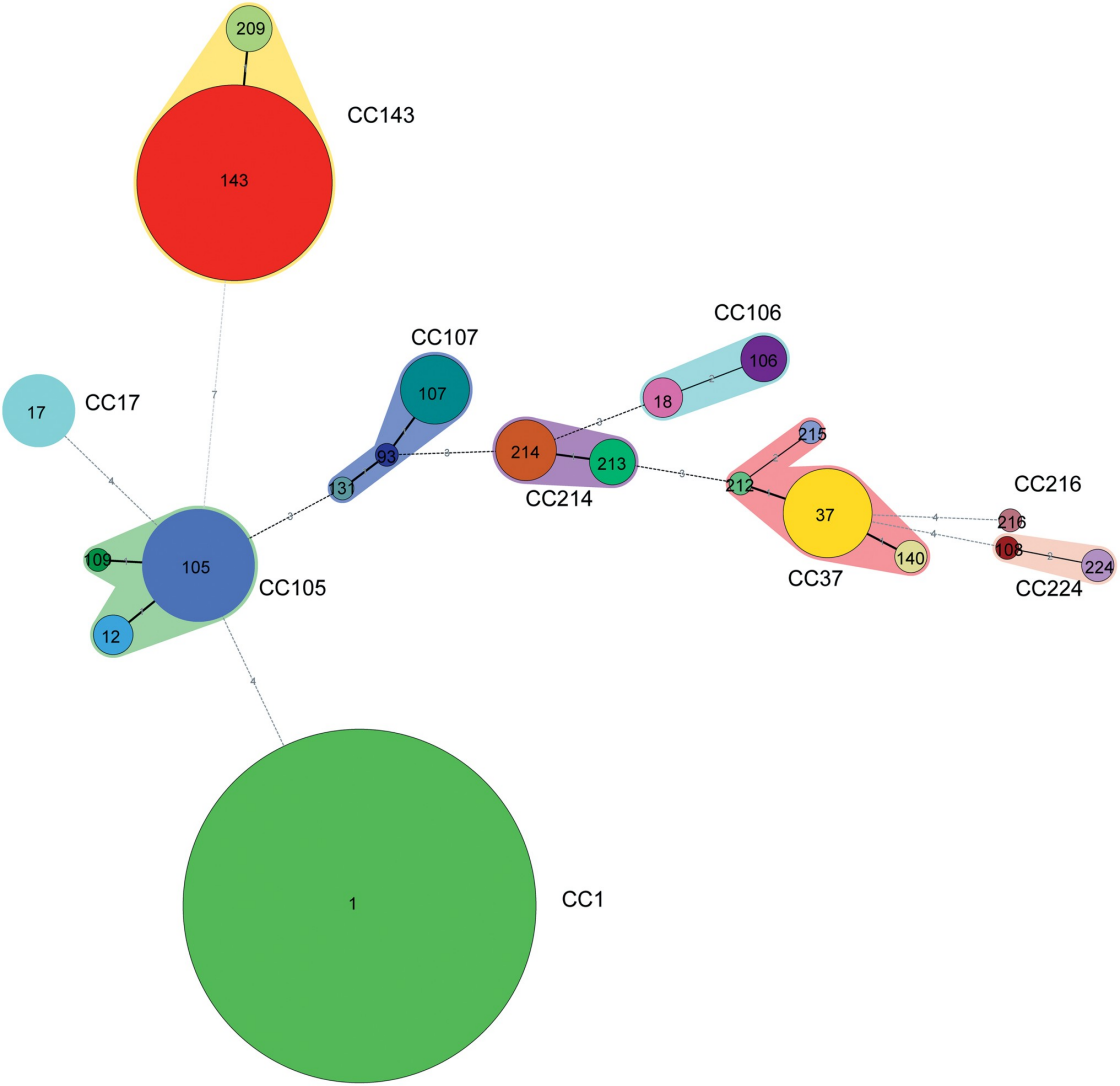
Minimum spanning tree analysis of 401 pathogenic *Leptospira* strains of Jiangxi. The size of the circle is proportional to the number of strains and the color indicates the diverse serogroup. The digits on the lines between two circles represent the different number of two STs. The shading surrounding the STs simply links STs within the same clonal complex (CCs). Ten CCs including (CC143, CC105, CC37, CC107, CC214, CC106, CC17, CC1, CC216 and CC224) were identified.

Minimum spanning tree analysis revealed 3 singletons (CC1, CC17 and CC216) and seven main CCs (CC143, CC105, CC37, CC107, CC214, CC106 and CC224) (Fig 2 and S1 Table). The most abundant CC was the Singleton CC1, with 235 ST1 isolates. The second most abundant, CC143, contained 76 isolates and was subdivided into two STs (ST143 and ST209), followed by CC105 (28 isolates in 3 STs) and CC37 (19 isolates in 4 STs). There was no coexistence of different species within a CC. As expected, only CC143 was classified as *L*. *borgpetersenii*, whereas the remaining six CCs and 3 singletons were classified as *L*. *interrogans*, showing no coexistence of different species within the same CC (Fig 2). Fisher’s exact test revealed highly significant differences in the distribution of leptospiral STs prevalence across collection locations of the 394 animal-associated strains (P < 0.001) (S9 Table). Based on the minimum spanning trees color-coded by monitoring sites, significant geographic variations in the distribution of dominant STs were also found: ST1 was the most prevalent ST in Shangrao (112/140) and Fuliang (123/136); ST143 was the most prevalent in Shangyou (46/52) and Longnan (19/26), but was also present in Shangrao; ST105 was the most prevalent in Shanggao (19/41); and ST209 was the most prevalent in Xinjian (4/6) (S2 Fig). The four most predominant genotypes, ST1, ST143, ST105 and ST37, were temporally (between 2002 and 2014) and geographically diverse (2 or 3 cities).

Fisher’s exact test revealed highly significant differences in the distribution of leptospiral ST prevalence across host species among the 394 animal-associated strains (P < 0.001) (S10 Table). From the Minimum spanning trees color-coded by animal host, significant differences in the distribution of leptospiral ST prevalence across host species were also found: ST1 and ST17 dominated in *A*. *agrarius* (86.9% and 80.0%, respectively), ST37 in dogs (86.7%), ST143 in *R*. *rattoides* (43.1%) and ST105 in *R*. *rattoides* (33.3%) (S3 Fig). Interestingly, ST105 strains were isolated from diverse sources including humans and a wide range of different animal hosts (*A*. *agrarius*, dogs, cattle, *Mus musculus*, *R*. *rattoides*, *R*. *norvegicus* and *R*. *nigromaculata*), indicating potential animal-to-human transmission.

From the minimum spanning trees color-coded by serogroup, each different serogroup corresponded to a special ST, except ST37 (S4 Fig). ST37 was related to serogroups of Canicola and Hebdomadis.

The Kruskal-Wallis chi-squared test showed a significant difference in the proportion of leptospiral ST prevalence in different years among the 394 animal-associated strains (χ^2^ = 84.7, df = 9, P < 0.001) (S11 Table). ST1, ST143 and ST105, as the prevalent STs, were present in nearly every year during the 2005–2015 period, with the exception of 2005 and 2014. ST17 was present in 2006–2009 and 2012. ST37 was present in 2006–2007, 2009 and 2011. Among the 401 isolates, the diversity of STs isolated annually was relatively low later in the survey period. Only two STs, ST107 and ST143, were found in 2005. An average of 5–14 different STs were found yearly between 2006 and 2011, but only 3 or 4 different STs were found each year between 2012 and 2015.

**Fig 3:**
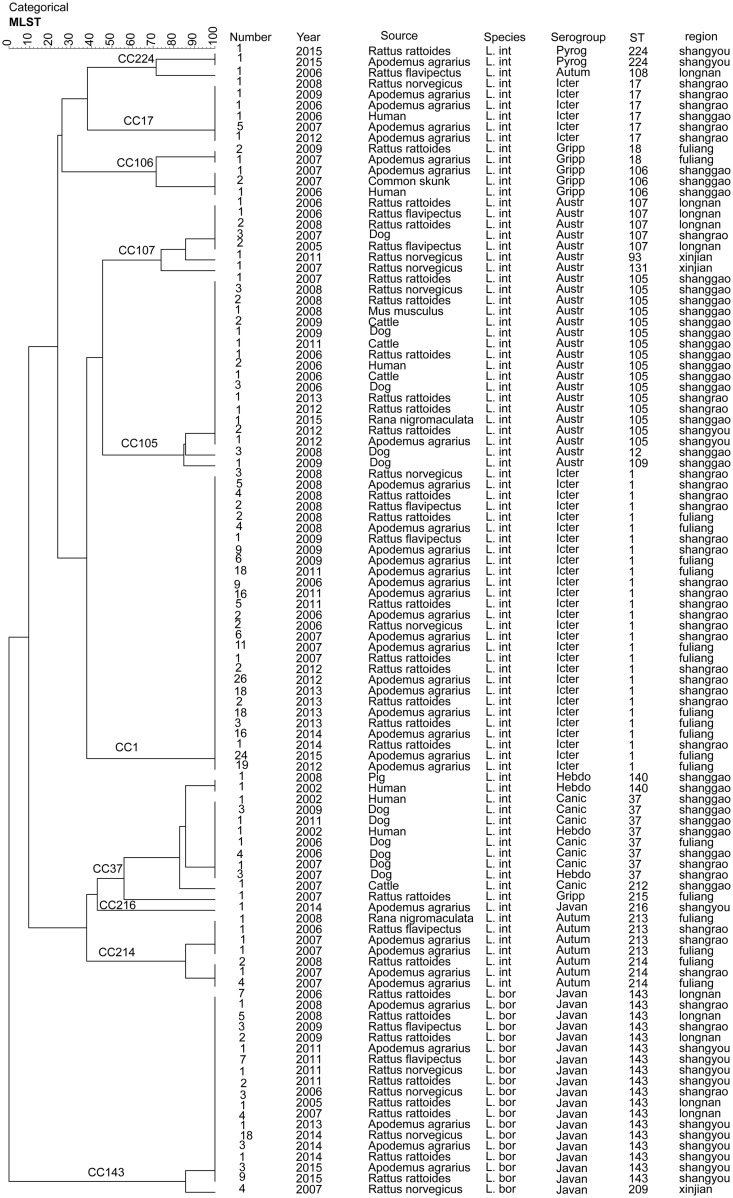
UPGMA dendrogram indicating the diversity of 401 Chinese pathogenic *Leptospira* strains of Jiangxi determined by MLST analysis. Groups were defined by a similarity of 60%. The dendrogram displays that the 401 Chinese *Leptospira* strains belonged to seven major clades and the remaining isolates were dispersed as unrelated singletons. (Austr: Australis, Autum: Autumnalis, Canic: Canicola, Gripp: Grippotyphosa, Hebdo: Hebdomadis, Icter: Icterohaemorrhagiae, Javan: Javanica, Pyrog: Pyrogenes, *L*. *int*: *L*. *interrogans*. *L*. *bor*: *L*. *borgpetersenii*).

The UPGMA dendrogram of the 401 isolates showed a relatively similar clustering patterns as determined using MST analysis (Figs 2 and 3). Seven main clades (CC143, CC105, CC37, CC107, CC214, CC106 and CC224) were generated and the remaining isolates were dispersed among three unrelated singletons (CC1, CC17 and CC216) (Fig 3). The majority of our isolates (58.6%, 235/401) belonged to the singleton CC1, followed by 76 isolates belonged to ST143 and ST209 of CC143.

The UPGMA dendrogram of STs showed that some isolates from humans and animals clustered closely together (Fig 3), such as ST17 (humans, *A*. *agrarius* and *R*. *norvegicus*), ST106 (humans, *A*. *agrarius* and skunk), ST105 (humans, *A*. *agrarius*, dogs, cattle, *M*. *musculus musculu*s, *R*. *rattoides*, *R*. *norvegicus* and *R*. *nigromaculata*), ST140 (humans and pigs) and ST37 (humans and dogs), For example, one clinical ST17 strain from 2006 and 9 rat-associated ST17 strains (*A*. *agrarius* and *R*. *norvegicus*) collected later clustered together. Another clinical ST106 strain also closely clustered with 3 ST106 strains from 1 *A*. *agrarius* and 2 common skunks in Shanggao. Moreover, 13 ST37 strains from dogs in 2006, 2007, 2009, 2011 and 2 clinical strains in Shanggao distributed into one cluster (Fig 3), indicating multiple animal-to-human transmission patterns of *L*. *interrogans*.

## Discussion

To our knowledge, this is the first large-scale study investigating distribution and abundance of pathogenic *Leptospira* strains isolated from small animal populations and their *Leptospira* carriage rates in Jiangxi Province from 2002 to 2015. Moreover, we provided the first description of circulating *Leptospira* serogroups, species and genotypes in humans and potential small animal reservoirs and/or carriers of leptospirosis in Jiangxi. Based on serological and microbiological methods in our study, we revealed that the proportion of infectious leptospiral prevalence in this study varied significantly across serogroups, species and STs, which was generally related to geography and the host species. The application of an epidemiological approach that includes ecological and evolutionary investigations can help provide insights into potential disease factors that may influence the morbidity rates of leptospirosis.

Host associations and biogeography are two important factors that may have direct effects on the pathogen transmission patterns of leptospirosis around the world. To our knowledge, this is the first study that takes into account the relationship between *Leptospira* prevalence proportion and the geographical location, collection time, and host species over a period of 14 years in Jiangxi Province, China. The statistical tests showed significant differences in the distribution of leptospiral prevalence across different host species, collection years and locations (S3–S11 Tables). Among the 401 Jiangxi isolates, serological typing revealed that the three predominant serogroups, Icterohaemorrhagiae, Javanica and Australis, were responsible for leptospirosis in Jiangxi Province from 2002 to 2014. Host-specific associations by serogroup existed at some degree in this study. Some serogroups were primarily associated with one host species; for example, *Rattus spp*. was the main carriers of Icterohaemorrhagiae, while dogs were the main carriers of Canicola. This was consistent with leptospiral serogroup-host associations that have been generally observed worldwide. For example, *Rattus spp*. are known carriers of Icterohaemorrhagiae [6, 19]. Differences in geographic distribution of serovars between ecological zones on Tutuila revealed that the three dominant serovars had different host species that live in different environments, which supports the hypothesis that environmental factors play an important role in the transmission dynamics of serovars [20].

This study suggests high diversity of pathogenic *Leptospira*, as the widespread and prevalent species, *L*. *interrogan*, and *L*. *borgpetersenii*, have been reported in humans and potentially identified in small animal reservoirs in China. This observation is consistent with previous genotyping investigations indicating that *L*. *interrogans*, *L*. *borgpetersenii* and *L*. *kirschneri* are the most abundant species circulating worldwide [21]. One previous study demonstrated that *L*. *interrogans* and *L*. *kirschneri* were identified using 16S rRNA gene sequencing and MLSA (multilocus sequence analysis) among 51 strains isolated from a variety of sources and geographical areas in France [22]. *L*. *interrogans* was found in several outbreaks in Brazil, Cambodia, Lao PDR and Thailand [21, 23–25]. In our present study, a significant difference in the proportions of leptospiral species prevalence across different host species/collection locations was found. *L*. *interrogans* was the most prevalent species, identified in all seven humans and 80.7% of animals screened in Jiangxi. As the most prevalent species in Jiangxi, *L*. *interrogans* was widely isolated from *A*. *agrarius*, *R*. *rattus*, *R*. *norvegicus*, *R*. *flavipectus*, dogs, cattle, *R*. *nigromaculata*, *M*. *musculus musculus*, pigs, skunks and humans. Among these hosts, *A*. *agrarius* was the most abundantly trapped host, whereas, *L*. *borgpetersenii* was isolated from *A*. *agrarius*, *R*. *rattus*, *R*. *norvegicus*, and *R*. *flavipectus*. This is not consistent with a previous report revealing host-species association in pathogenic *Leptospira* species in other countries [26]. In contrast with previous surveys where carriage rates ranged from 11% to 80.3% when based on culture isolation, the carriage rate in rats detected in this investigation (9.35%) was lower [27–29]. Our results give the first direct confirmation that *A*. *agrarius* infected with the same prevalent serogroups of Icterohaemorrhagiae was recognized as the major potential animal reservoir of *L*. *interrogans*, the same most prevalent species identified from human leptospirosis patients in China. This observation is consistent with the fact that *L*. *interrogans* and *L*. *borgpetersenii* are commonly associated with rodents worldwide [25, 30, 31].

Leptospiral diversity and prevalence can be affected by a number of environmental factors. The distribution of *L*. *interrogans* and *L*. *borgpetersenii* associated with special geographic regions in Jiangxi was also confirmed in this study. *L*. *interrogans* and *L*. *borgpetersenii*, as the two most prevalent *Leptospira* species, may have different epidemiological transmission patterns. It has been reported that *L*. *interrogans* infection in rodents is restricted to humid habitats, while *L*. *borgpetersenii* infection occurs in both humid and dry climates [25, 32]. Leptospiral diversity may be due to the difference of special geographic regions. In this study, Shangrao, Fuliang and Shanggao (Habitat B) were in the northeastern hilly plain area and have relatively humid habitats characteristic of Jiangxi, while Shangyou and Longnan (Habitat C) are located in the southwestern Jiangxi mountain region and have relatively dry habitats; Xinjian (Habitat A) is located in the northern lower Poyang Lake flat area. The differences in climate, geomorphology and altitude among these areas may influence leptospiral clade diversity. How host-pathogen interactions, ecosystems and geographical factors influence the community ecology of a pathogen is unclear.

Among wild animals, rodents are the primary prevalence maintenance hosts for *Leptospira* spp. and may transfer infection to livestock, small wild animals and humans [33]. In our study, the prevalent serogroups/STs of the strains isolated from patients and possible animal reservoirs display a high similarity in Jiangxi Province (Fig 3), indicating the close transmission relationship of these *Leptospira*. For example, CC37, as one of the main clone complexes, was common in pigs, dogs, cattle, and *R*. *rattoides*, as well as humans, in Shanggao, Shangrao and Fuliang between 2002 and 2011. It was shown that dogs, pigs and cattle, as well as rodents, are also important reservoirs for the transmission of *Leptospira* to humans. It was reported that most of the leptospirosis cases in China occurred from July to December, with a peak in September [34]. This is the period of rice planting and harvest in Southern China. Farmers can be infected through direct contact with infected domestic animals (pigs, dogs and cattle) or wild animals (rodents, the common skunks, *R*. *nigromaculata* and so on) or through indirect contact with water and soil contaminated by the urine of infected animals. The results in our study may assist in efforts to track the potential transmission source of leptospirosis outbreaks and to establish a better control program against leptospirosis in different epidemic regions. Further studies are needed to determine whether the prevalence of leptospirosis in Jiangxi Province is similar to that in other countries worldwide.

Five of the most prevalent STs, ST1, ST143, ST105, ST37 and ST17, were identified as longterm and ubiquitous virulent strains throughout Jiangxi Province. Compared to the predominant ST1 widely distributed between host clades (*A*. *agrarius*, *R*. *norvegicus*, *R*. *rattoides* and *R*. *flavipectus*) and geographic locations (Shangrao, and Fuliang) during 2006–2015, ST37 was mainly distributed among dogs and humans in Fuliang, Shanggao and Shangrao in 2002, 2006–2007, 2009 and 2011. Similarly, ST17 was distributed between *A*. *agrarius*, *R*. *norvegicus* and humans in Jiangxi in 2006–2009 and 2012, whereas ST105 was distributed between wider spectrum of host clades (*A*. *agrarius*, dogs, cattle, *M*. *musculus*, *R*. *nigromaculata*, *R*. *norvegicus* and *R*. *rattoides*) during 2006–2013 and 2015. ST143, as the most predominant genotype ST in Jiangxi, was also reported in Malaysia [35]. The different isolation locations may be the key factor in the diversity of circulating *Leptospira spp*. Shangyou and Longnan (Habitat C) were located in the south Jiangxi mountain region, with elevations ranging from 1000–1600 meters above sea level. The differences in climate, geomorphology and altitude among these locations may influence leptospiral clade diversity. In our study, variation of the serogroups or STs reflected the features of *Leptospira* in Jiangxi. Thaipadungpanit et al. reported that ST34 was the most frequent genotype in 101 *L*. *interrogans* strains in Thailand in 2007 [21]. Caimi et al. demonstrated that ST37 was the main genotype in 18 isolates in Argentina [24]. ST17 was identified in 90 strains of serogroup Icterohaemorrhagiae in Sao Paulo [36]. Five common STs, ST37, ST17, ST 199, ST110, and ST146, were reported to have a longterm and ubiquitous distribution in Russia [37]. ST37, ST118 and ST119 were isolated from dogs in Japan [38]. ST110, ST50, ST143 and ST242 were reported in small mammals in Malaysia [39]. Therefore, these prevalent STs (ST17, ST37 and ST143) reported in China are also the same prevalent STs in the rest of the world. At the same time, the genetic diversity of *Leptospira* in China is generally different from that observed in other countries, suggesting a high degree of diversity of circulating *Leptospira spp* worldwide. ST17 and ST37 were found to be the most globally prevalent strains of pathogenic *Leptospira* circulating in a wide geographic region that includes China. These specific dominant epidemic strains, such as ST17 and ST37, may have selective advantages in the environment or in possible animal reservoirs that have allowed them to survive and become unusually geographically widespread. However, some STs appear to be concentrated in specific geographic regions: ST1, as the most prevalent ST, has only been reported in Chinese strains; ST145, the most prevalent ST in India, is not distributed worldwide [40]. *L*. *interrogans*, *L*. *kirschneri* ST117 and *L*. *kirschneri* ST110 were present in small mammals at all three sites surveyed in Germany [41]. Generally, there is a complex population structure and biased distribution of genotypes of *Leptospira* isolates worldwide. The findings of this study highlight the importance of understanding the epidemiology and ecology of *Leptospira* worldwide.

Here, our focus on pathogenic *Leptospira* using serogroup identification, 16S rRNA gene sequencing and MLST analysis for phylogenetic analysis has led to a better understanding of diversity of *Leptospira*. MLST provides evidence that the diversity of STs is very high in China. The results may be useful in developing a strategy and guidelines for the prevention and control of leptospirosis in China. While, phylogenetic analysis of more globally *Leptospira* isolates is necessary, we nonetheless believe that our present study provides a blueprint for further phylogenetic studies.

## Supporting information

S1 Table 401 pathogenic *Leptospira* isolates of Jiangxi used in this study. (XLSX)

S2 Table 16S rRNA gene sequences of 20 Leptospira reference species, Leptonema illini NCTC 11301T and Turneriella parva NCTC 11395T obtained from GenBank database. (DOCX)

S3 Table Species distribution of the 394 animal associated-Leptospira spp. among the different captured wild small animals in Jiangxi. (XLSX)

S4 Table Species distribution of the 394 animal associated-Leptospira spp. in different collected locations in Jiangxi. (XLSX)

S5 Table Species distribution of the 394 animal associated-Leptospira spp. in different collected years in Jiangxi. (XLSX)

S6 Table Serogroups distribution of the 394 animal associated-Leptospira spp. in different collected locations in Jiangxi. (XLSX)

S7 Table Serogroups distribution of the 394 animal associated-Leptospira spp. among the different captured wild small animals in Jiangxi. (XLSX)

S8 Table Serogroups distribution of the 394 animal associated-Leptospira spp. in different collected years in Jiangxi. (XLSX)

S9 Table Sequence types distribution of the 394 animal associated-Leptospira spp. in different collected locations in Jiangxi. (XLSX)

S10 Table Sequence types distribution of the 394 animal associated-Leptospira spp. among the different captured wild small animals in Jiangxi. (XLSX)

S11 Table Sequence types distribution of the 394 animal associated-Leptospira spp. in different collected years in Jiangxi. (XLSX)

S1 Fig Phylogenetic analysis based on 16SrRNA gene sequencing for the 401 pathogenic Leptospira strains in Jiangxi. The dendrogram displays that the 401 *Leptospira* strains were identified to two *Leptospira* species of *L*. *interrogans* and *L*. *borgpetersenii*. Each species is labeled as follows: (*L*. *interrogans*; *L*. *borgpetersenii*; *L*. *kirschneri*; *L*. *alstonii*; *L*. *noguchii; L*. *santarosai*; *L*. *weilii*; *L*. *kmetyi*; *L*. *alexanderi; L*. *inadai; L*. *wolffii; L*. *broomii; L*. *licerasiae; L*. *fainei; L*. *biflexa; L*. *wolbachii; L*. *meyeri; L*. *yanagawae; L*. *terpstrae; L*. *vanthielii; T*. *parva* and *L*. *illini*).
(TIF)

S2 Fig Minimum spanning trees color-coded by monitoring sites of 401 pathogenic Leptospira strains in Jiangxi Province in China. (TIF)

S3 Fig Minimum spanning trees color-coded by animal hosts of 401 pathogenic Leptospira strains in Jiangxi Province in China. (TIF)

S4 Fig Minimum spanning trees color-coded by serogroups of 401 pathogenic Leptospira strains in Jiangxi Province in China. (TIF)

S1 Checklist STROBE Checklist. (DOC)

## References

[1] Adler B. History of leptospirosis and leptospira. Curr Top Microbiol Immunol. 2015;387:1–9. 10.1007/978-3-662-45059-8_1 . 25388129

[2] Costa F, Wunder EA Jr., De Oliveira D, Bisht V, Rodrigues G, Reis MG, et al Patterns in Leptospira Shedding in Norway Rats (Rattus norvegicus) from Brazilian Slum Communities at High Risk of Disease Transmission. PLoS Negl Trop Dis. 2015;9(6):e0003819 10.1371/journal.pntd.0003819 ; 26047009 PMC4457861

[3] Picardeau M. Virulence of the zoonotic agent of leptospirosis: still terra incognita? Nat Rev Microbiol. 2017;15(5):297–307. 10.1038/nrmicro.2017.5 . 28260786

[4] Hu W, Lin X, Yan J. Leptospira and leptospirosis in China. Curr Opin Infect Dis. 2014;27(5):432–6. 10.1097/QCO.0000000000000097 . 25061933

[5] Dietrich M, Wilkinson DA, Soarimalala V, Goodman SM, Dellagi K, Tortosa P. Diversification of an emerging pathogen in a biodiversity hotspot: Leptospira in endemic small mammals of Madagascar. Mol Ecol. 2014;23(11):2783–96. Epub 2014/05/03. 10.1111/mec.12777 . 24784171

[6] Vinetz. JM, Wilcox. BA, Aguirre. A, Gollin. LX, Katz. AR, Fujioka. RS, et al Beyond Disciplinary Boundaries: Leptospirosis as a Model of Incorporating Transdisciplinary Approaches to Understand Infectious Disease Emergence. EcoHealth. 2005;2(4):291–306.

[7] Haake DA, Levett PN. Leptospirosis in humans. Curr Top Microbiol Immunol. 2015;387:65–97. Epub 2014/11/13. 10.1007/978-3-662-45059-8_5 ; 25388133 PMC4442676

[8] Ellis WA. Animal leptospirosis. Curr Top Microbiol Immunol. 2015;387:99–137. Epub 2014/11/13. 10.1007/978-3-662-45059-8_6 . 25388134

[9] Zhang C, Wang H, Yan J. Leptospirosis prevalence in Chinese populations in the last two decades. Microbes Infect. 2012;14(4):317–23. 10.1016/j.micinf.2011.11.007 . 22155621

[10] Wang YL, Qin JH, Zhang CC, Guo XK, Jiang XG, He P. An outbreak of leptospirosis in Lezhi County, China in 2010 may possibly be linked to rainfall. Biomed Environ Sci. 2014;27(1):56–9. Epub 2014/02/21. 10.3967/bes2014.016 . 24553376

[11] Luo HS, Xu HD, Hu JG. Geographic epidemiology research of leptospirosis in JiangXi Province. Zhong Hua Liu Xing Bing Xue Za Zhi. 1995;16(4):93–103. Epub 1995.

[12] Morand. S, Bordes. F, Blasdel. K, Pilosof. S, Cornu. JF, Chaisiri. K, et al Assessing the distribution of disease-bearing rodents in human-modified tropical landscapes. Journal of Applied Ecology. 2015;52(3):784–94. Epub 2015 February. 10.1111/1365-2664.12414

[13] Pages M, Chaval Y, Herbreteau V, Waengsothorn S, Cosson JF, Hugot JP, et al Revisiting the taxonomy of the Rattini tribe: a phylogeny-based delimitation of species boundaries. BMC Evol Biol. 2010;10:184 Epub 2010/06/23. 10.1186/1471-2148-10-184 ; 20565819 PMC2906473

[14] Ahmad SN, Shah S, Ahmad FM. Laboratory diagnosis of leptospirosis. J Postgrad Med. 2005;51(3):195–200. Epub 2005/12/08. . 16333192

[15] Morey RE, Galloway RL, Bragg SL, Steigerwalt AG, Mayer LW, Levett PN. Species-specific identification of Leptospiraceae by 16S rRNA gene sequencing. Journal of clinical microbiology. 2006;44(10):3510–6. 10.1128/JCM.00670-06 . 17021075 PMC1594759

[16] Smythe L, Adler B, Hartskeerl RA, Galloway RL, Turenne CY, Levett PN. Classification of Leptospira genomospecies 1, 3, 4 and 5 as Leptospira alstonii sp. nov., Leptospira vanthielii sp. nov., Leptospira terpstrae sp. nov. and Leptospira yanagawae sp. nov., respectively. International journal of systematic and evolutionary microbiology. 2013;63(Pt 5):1859–62. 10.1099/ijs.0.047324-0 . 22984140

[17] Boonsilp S, Thaipadungpanit J, Amornchai P, Wuthiekanun V, Bailey MS, Holden MT, et al A single multilocus sequence typing (MLST) scheme for seven pathogenic Leptospira species. PLoS Negl Trop Dis. 2013;7(1):e1954 10.1371/journal.pntd.0001954 ; 23359622 PMC3554523

[18] John MC. Software for Data Analysis: Programming with R. Springer 2008.

[19] Wong M, Katz AR, Li D, Wilcox BA. Leptospira infection prevalence in small mammal host populations on three Hawaiian islands. Am J Trop Med Hyg. 2012;87(2):337–41. 10.4269/ajtmh.2012.12-0187 ; 22855767 PMC3414573

[20] Lau CL, Skelly C, Smythe LD, Craig SB, Weinstein P. Emergence of new leptospiral serovars in American Samoa—ascertainment or ecological change? BMC Infect Dis. 2012;12:19 10.1186/1471-2334-12-19 ; 22273116 PMC3305655

[21] Thaipadungpanit J, Wuthiekanun V, Chierakul W, Smythe LD, Petkanchanapong W, Limpaiboon R, et al A dominant clone of Leptospira interrogans associated with an outbreak of human leptospirosis in Thailand. PLoS Negl Trop Dis. 2007;1(1):e56 10.1371/journal.pntd.0000056 ; 17989782 PMC2041815

[22] Leon A, Pronost S, Fortier G, Andre-Fontaine G, Leclercq R. Multilocus sequence analysis for typing Leptospira interrogans and Leptospira kirschneri. J Clin Microbiol. 2010;48(2):581–5. 10.1128/JCM.00543-09 ; 19955271 PMC2815645

[23] Ko AI, Galvao Reis M, Ribeiro Dourado CM, Johnson WD Jr., Riley LW,. Urban epidemic of severe leptospirosis in Brazil. Salvador Leptospirosis Study Group. Lancet. 1999;354(9181):820–5. 10.1016/s0140-6736(99)80012-9 . 10485724

[24] Caimi K, Varni V, Melendez Y, Koval A, Brihuega B, Ruybal P. A combined approach of VNTR and MLST analysis: improving molecular typing of Argentinean isolates of Leptospira interrogans. Mem Inst Oswaldo Cruz. 2012;107(5):644–51. 10.1590/s0074-02762012000500011 . 22850955

[25] Cosson JF, Picardeau M, Mielcarek M, Tatard C, Chaval Y, Suputtamongkol Y, et al Epidemiology of leptospira transmitted by rodents in southeast Asia. PLoS Negl Trop Dis. 2014;8(6):e2902 10.1371/journal.pntd.0002902 ; 24901706 PMC4046967

[26] Dietrich M, Gomard Y, Lagadec E, Ramasindrazana B, Le Minter G, Guernier V, et al Biogeography of Leptospira in wild animal communities inhabiting the insular ecosystem of the western Indian Ocean islands and neighboring Africa. Emerg Microbes Infect. 2018;7(1):57 10.1038/s41426-018-0059-4 ; 29615623 PMC5883017

[27] de Faria MT, Calderwood MS, Athanazio DA, McBride AJ, Hartskeerl RA, Pereira MM, et al Carriage of Leptospira interrogans among domestic rats from an urban setting highly endemic for leptospirosis in Brazil. Acta Trop. 2008;108(1):1–5. 10.1016/j.actatropica.2008.07.005 ; 18721789 PMC2596941

[28] Perez J, Brescia F, Becam J, Mauron C, Goarant C. Rodent abundance dynamics and leptospirosis carriage in an area of hyper-endemicity in New Caledonia. PLoS Negl Trop Dis. 2011;5(10):e1361 10.1371/journal.pntd.0001361 ; 22039557 PMC3201910

[29] Benacer D, Mohd Zain SN, Sim SZ, Mohd Khalid MK, Galloway RL, Souris M, et al Determination of Leptospira borgpetersenii serovar Javanica and Leptospira interrogans serovar Bataviae as the persistent Leptospira serovars circulating in the urban rat populations in Peninsular Malaysia. Parasit Vectors. 2016;9:117 10.1186/s13071-016-1400-1 ; 26927873 PMC4772511

[30] Biscornet L, Dellagi K, Pages F, Bibi J, de Comarmond J, Melade J, et al Human leptospirosis in Seychelles: A prospective study confirms the heavy burden of the disease but suggests that rats are not the main reservoir. PLoS Negl Trop Dis. 2017;11(8):e0005831 10.1371/journal.pntd.0005831 ; 28846678 PMC5591009

[31] Guernier V, Richard V, Nhan T, Rouault E, Tessier A, Musso D. Leptospira diversity in animals and humans in Tahiti, French Polynesia. PLoS Negl Trop Dis. 2017;11(6):e0005676 10.1371/journal.pntd.0005676 ; 28658269 PMC5507467

[32] Bulach DM, Zuerner RL, Wilson P, Seemann T, McGrath A, Cullen PA, et al Genome reduction in Leptospira borgpetersenii reflects limited transmission potential. Proc Natl Acad Sci U S A. 2006;103(39):14560–5. 10.1073/pnas.0603979103 ; 16973745 PMC1599999

[33] Faine S, Adler B, Bolin C, Perolat P. Leptospira and Leptospirosis. Austrialia, Melbourne, MediSci. 1999:83–6.

[34] Zhang C, Li Z, Xu Y, Zhang Y, Li S, Zhang J, et al Genetic diversity of Leptospira interrogans circulating isolates and vaccine strains in China from 1954–2014. Hum Vaccin Immunother. 2019;15(2):381–7. Epub 2018/09/28. 10.1080/21645515.2018.1528839 ; 30260259 PMC6422446

[35] Benacer D, Mohd Zain SN, Ahmed AA, Mohd Khalid MK, Hartskeerl RA, Thong KL. Predominance of the ST143 and ST50 Leptospira clones in the urban rat populations of Peninsular Malaysia. J Med Microbiol. 2016;65(6):574–7. 10.1099/jmm.0.000262 . 27058766

[36] Romero EC, Blanco RM, Galloway RL. Analysis of multilocus sequence typing for identification of Leptospira isolates in Brazil. J Clin Microbiol. 2011;49(11):3940–2. 10.1128/JCM.01119-11 ; 21880969 PMC3209071

[37] Voronina OL, Kunda MS, Aksenova EI, Ryzhova NN, Semenov AN, Petrov EM, et al The characteristics of ubiquitous and unique Leptospira strains from the collection of Russian centre for leptospirosis. Biomed Res Int. 2014;2014:649034 10.1155/2014/649034 ; 25276806 PMC4167648

[38] Koizumi N, Muto MM, Akachi S, Okano S, Yamamoto S, Horikawa K, et al Molecular and serological investigation of Leptospira and leptospirosis in dogs in Japan. J Med Microbiol. 2013;62(Pt 4):630–6. 10.1099/jmm.0.050039-0 . 23264455

[39] Azhari NN, Ramli SNA, Joseph N, Philip N, Mustapha NF, Ishak SN, et al Molecular characterization of pathogenic Leptospira sp. in small mammals captured from the human leptospirosis suspected areas of Selangor state, Malaysia. Acta Trop. 2018;188:68–77. 10.1016/j.actatropica.2018.08.020 . 30145261

[40] Kanagavel M, Princy Margreat AA, Arunkumar M, Prabhakaran SG, Shanmughapriya S, Natarajaseenivasan K. Multilocus sequence typing (MLST) of leptospiral strains isolated from two geographic locations of Tamil Nadu, India. Infect Genet Evol. 2016;37:123–8. 10.1016/j.meegid.2015.11.008 . 26577860

[41] Obiegala A, Woll D, Karnath C, Silaghi C, Schex S, Essbauer S, et al Prevalence and Genotype Allocation of Pathogenic Leptospira Species in Small Mammals from Various Habitat Types in Germany. PLoS Negl Trop Dis. 2016;10(3):e0004501 10.1371/journal.pntd.0004501 ; 27015596 PMC4807814

