# Supplementary material for: Genetic characteristics of pathogenic Leptospira in wild small animals and livestock in Jiangxi Province, China, 2002–2015
Source: PLoS Negl Trop Dis. 2019 Jun 24;13(6):e0007513. doi: 10.1371/journal.pntd.0007513 (PMC6611636; doi:10.1371/journal.pntd.0007513)
Supplement: S2 Table — (DOCX) [file pntd.0007513.s002.docx]

Table S2. 16S rRNA sequences of 20 *Leptospira* species, *Turneriella parva* NCTC 11395T and *Leptonema illini* NCTC 11301T obtained from GenBank database

| **Clade** | **Species** | **Serovar** | **Strain** | **GenBank ID** |
| --- | --- | --- | --- | --- |
| **Pathogenic** | ***L. alstonii*** | **Sichuan** | **ATCC BAA-2439 ^T^** | **AY631881** |
| **Pathogenic** | ***L. santarosai*** | **Shermani** | **ATCC 43286 ^T^** | **AY631883** |
| **Pathogenic** | ***L. borgpetersenii*** | **Ballum** | **Mus 127** | **AY631884** |
| **Pathogenic** | ***L. noguchii*** | **Panama** | **ATCC 43288 ^T^** | **AY631886** |
| **Pathogenic** | ***L. interrogans*** | **Ballum** | **RGAT** | **AY631894** |
| **Pathogenic** | ***L. kirschneri*** | **Cynopteri** | **ATCC 49945 ^T^** | **AY631895** |
| **Pathogenic** | ***L. weilii*** | **Celledoni** | **ATCC 43285 ^T^** | **AY631877** |
| **Pathogenic** | ***L. alexanderi*** | **Manhao 3** | **ATCC 700520 ^T^** | **AY631880** |
| **Pathogenic** | ***L. kmetyi*** | **Not designated** | **Bejo Iso9 ^T^** | **AB279549** |
| **Intermediate** | ***L. inadai*** | **Lyme** | **Lyme 10 ^T^** | **AY631896** |
| **Intermediate** | ***L. broomii*** | **Not designated** | **ATCC BAA-1107 ^T^** | **AY796065** |
| **Intermediate** | ***L. wolffii*** | **Not designated** | **Khorat-H2 ^T^** | **EF025496** |
| **Intermediate** | ***L. licerasiae*** | **Not designated** | **ATCC BAA 1110 ^T^** | **EF612284** |
| **Intermediate** | ***L. fainei*** | **Hurstbridge** | **ATCC BAA-1107 ^T^** | **AY631885** |
| **Nonpathogenic** | ***L. biflexa*** | **Patoc** | **ATCC 23582 ^T^** | **AY631876** |
| **Nonpathogenic** | ***L. meyeri*** | **Ranarum** | **ATCC 43287 ^T^** | **AY631878** |
| **Nonpathogenic** | ***L. wolbachii*** | **Codice** | **ATCC 43284 ^T^** | **AY631879** |
| **Nonpathogenic** | ***L. yanagawae*** | **Saopaulo** | **ATCC 700523 ^T^** | **AY631882** |
| **Nonpathogenic** | ***L. terpstrae*** | **Hualin** | **ATCC 700639 ^T^** | **AY631888** |
| **Nonpathogenic** | ***L. vanthielii*** | **Holland** | **ATCC 700522 ^T^** | **AY631897** |
| **Other** | ***T. parva*** | **Parva** | **NCTC 11395 ^T^** | **AY293856** |
|  | ***L. illini*** | **Illini** | **NCTC 11301 ^T^** | **AY714984** |
