# Supplementary figures and images for: Genetic characteristics of pathogenic Leptospira in wild small animals and livestock in Jiangxi Province, China, 2002–2015
Source: PLoS Negl Trop Dis. 2019 Jun 24;13(6):e0007513. doi: 10.1371/journal.pntd.0007513 (PMC6611636; doi:10.1371/journal.pntd.0007513)

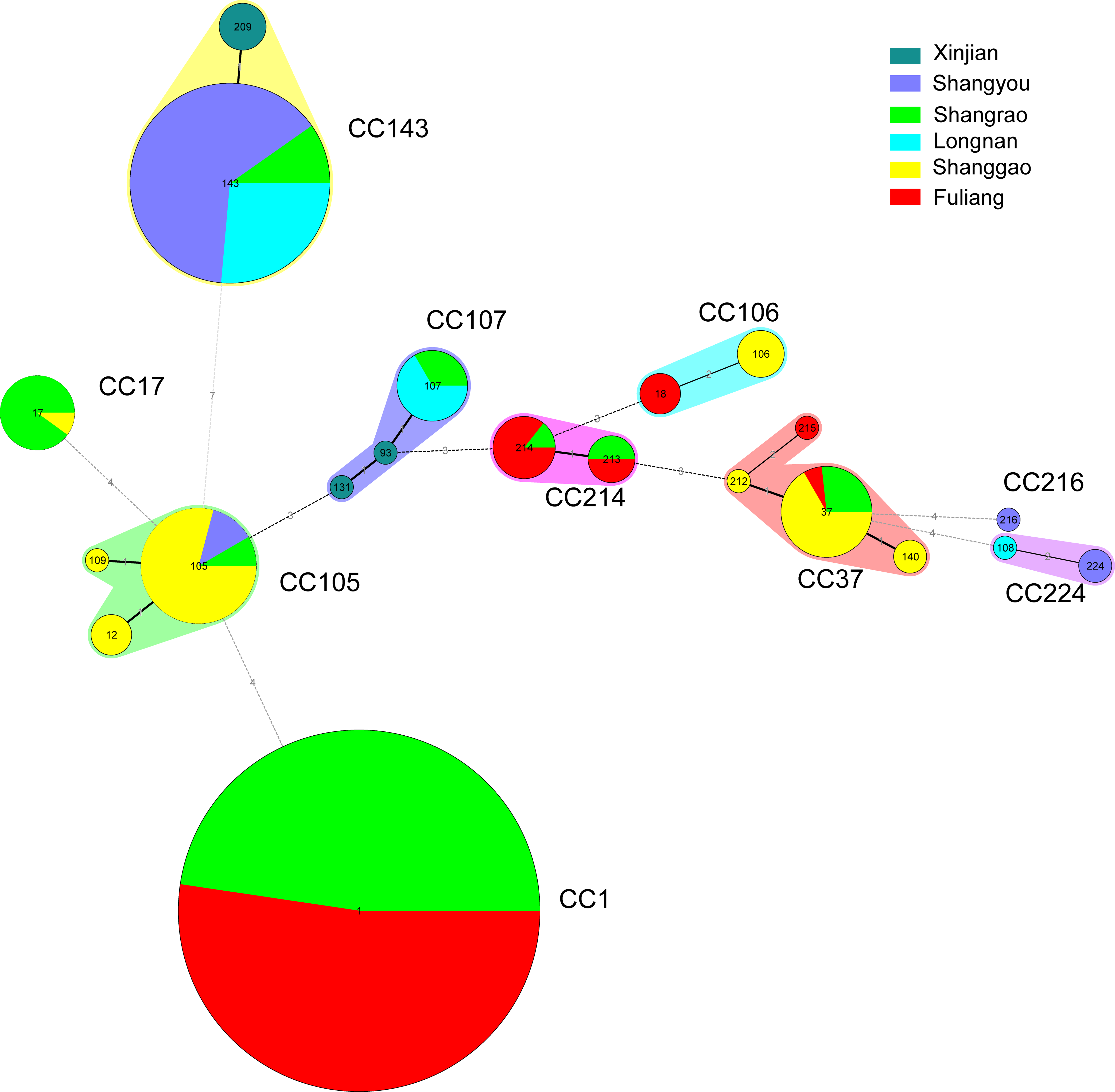

Supplement: S2 Fig — (TIF) [file pntd.0007513.s013.tif]

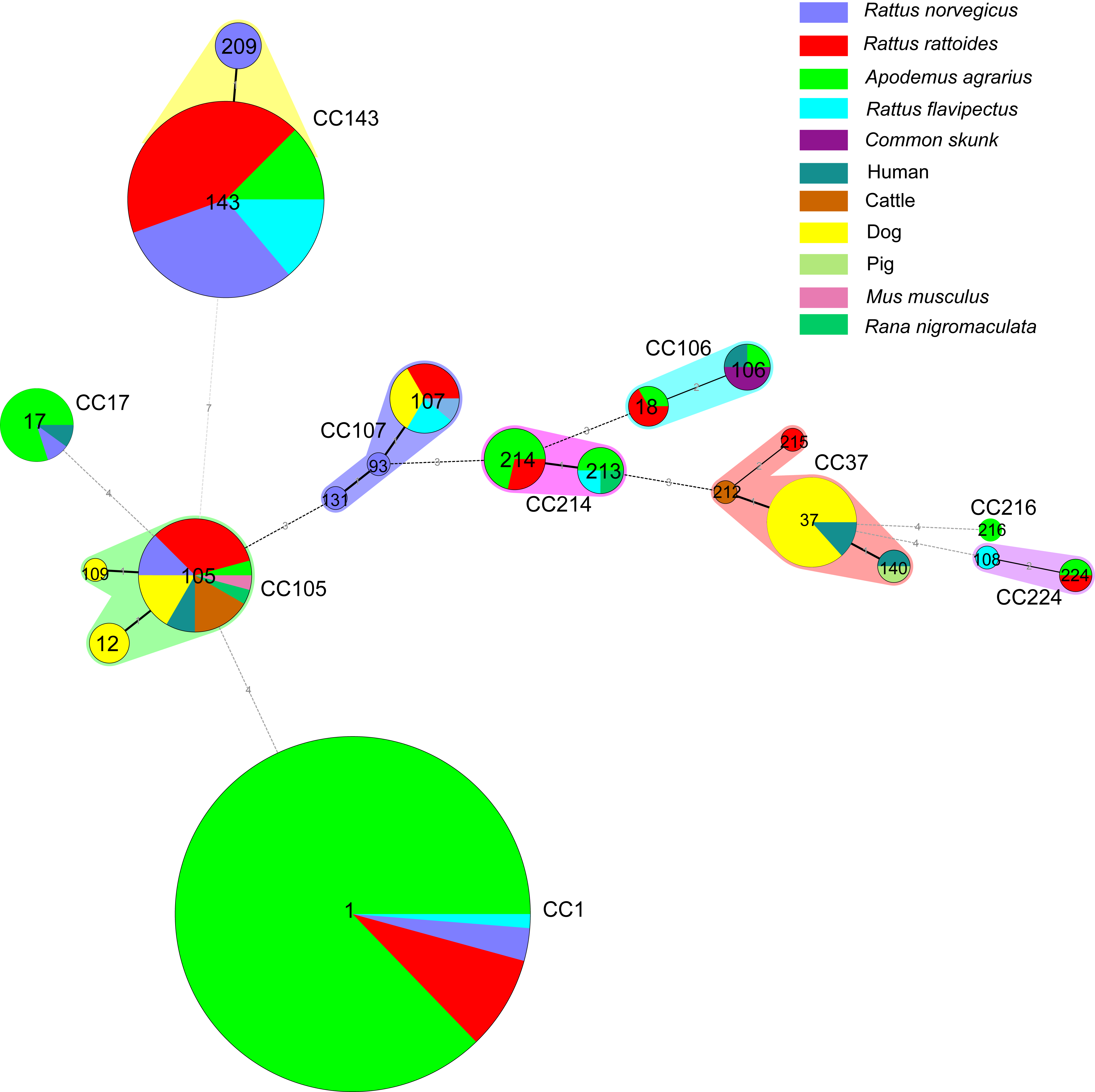

Supplement: S3 Fig — (TIF) [file pntd.0007513.s014.tif]

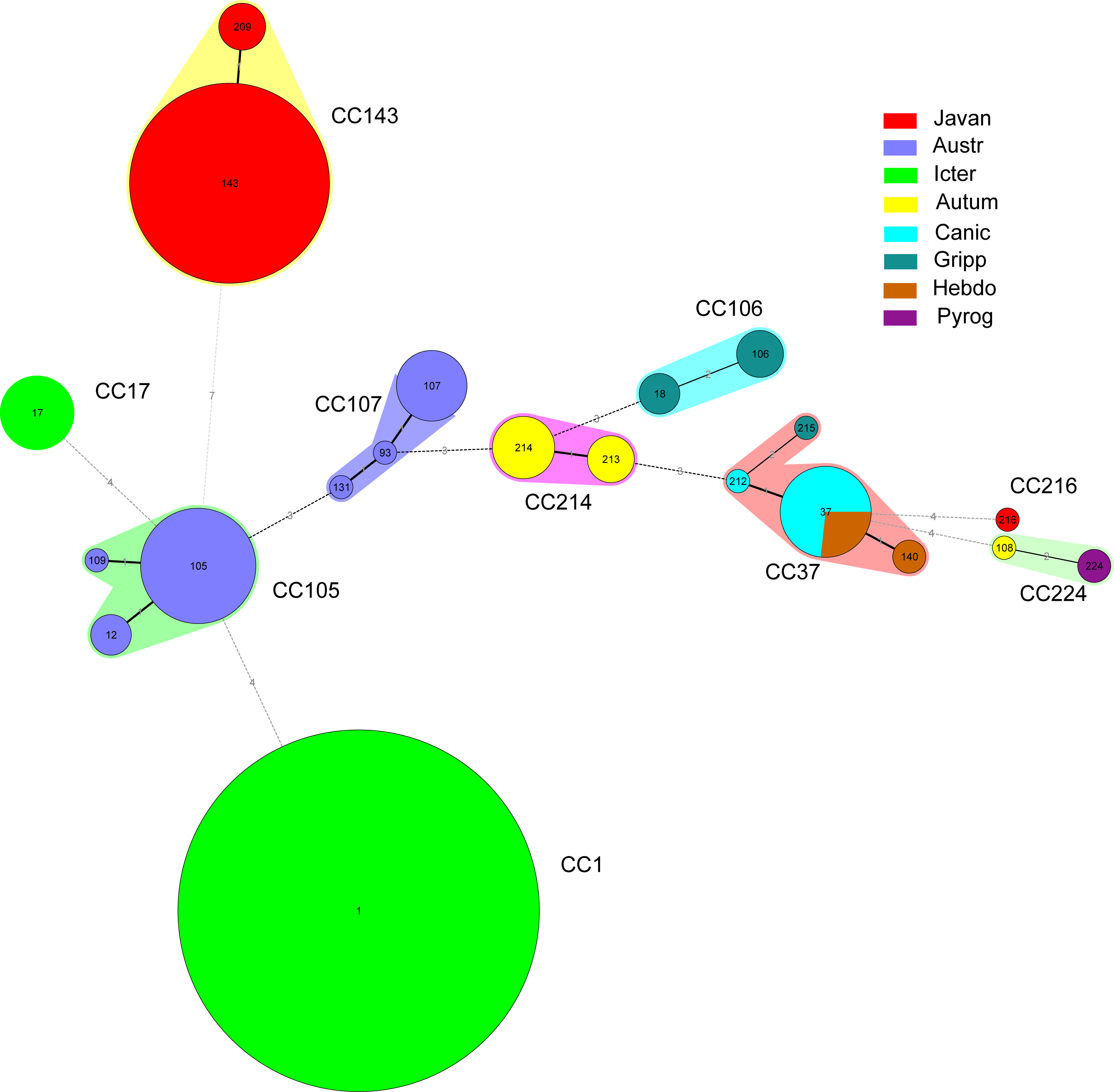

Supplement: S4 Fig — (TIF) [file pntd.0007513.s015.tif]
