# Supplementary material for: Genetic characteristics of pathogenic Leptospira in wild small animals and livestock in Jiangxi Province, China, 2002–2015
Source: PLoS Negl Trop Dis. 2019 Jun 24;13(6):e0007513. doi: 10.1371/journal.pntd.0007513 (PMC6611636; doi:10.1371/journal.pntd.0007513)
Supplement: S1 Checklist — (DOC) [file pntd.0007513.s016.doc]

STROBE Statement—checklist of items that should be included in reports of observational studies

|  | Item No | Recommendation |
| --- | --- | --- |
| **Title and abstract** | Line1-116 | (*a*) Indicate the study’s design with a commonly used term in the title or the abstract |
|  | | |
| **Introduction** | Line118-195 | State specific objectives, including any prespecified hypotheses |
|  | | |
| **Materials and Methods** | Line198-204 | Present key elements of study design early in the paper |
|  | | |
| **Results** | Line274-408 | (a) Report numbers of individuals at each stage of study—eg numbers potentially eligible, examined for eligibility, confirmed eligible, included in the study, completing follow-up, and analysed |
|  | | |
| **Discussion** | Line410-552 | Summarise key results with reference to study objectives |
|  |  |  |
| **Acknowledgments** | Line554-558 | Discuss limitations of the study, taking into account sources of potential bias or imprecision. Discuss both direction and magnitude of any potential bias |
| **Funding** | Line561-565 | Give the source of funding and the role of the funders for the present study and, if applicable, for the original study on which the present article is based |
|  |  |  |
| **Figure legends** | Line674-701 | Figure legends |
| **Supporting Information Legends** | Line704-726 | Supporting Information Legends |
|  |  |  |

*Give information separately for cases and controls in case-control studies and, if applicable, for exposed and unexposed groups in cohort and cross-sectional studies.

**Note:** An Explanation and Elaboration article discusses each checklist item and gives methodological background and published examples of transparent reporting. The STROBE checklist is best used in conjunction with this article (freely available on the Web sites of PLoS Medicine at http://www.plosmedicine.org/, Annals of Internal Medicine at http://www.annals.org/, and Epidemiology at http://www.epidem.com/). Information on the STROBE Initiative is available at www.strobe-statement.org.
